# Natural Hazard Susceptibility Assessment for Road Planning Using Spatial Multi-Criteria Analysis

**DOI:** 10.1007/s00267-017-0912-6

**Published:** 2017-08-18

**Authors:** Caroline S. J. Karlsson, Zahra Kalantari, Ulla Mörtberg, Bo Olofsson, Steve W. Lyon

**Affiliations:** 1 Division of Land and Water Resources Engineering, KTH Royal Institute of Technology, SE-100 44 Stockholm, Sweden; 2 Department of Physical Geography, Stockholm University, SE-106 91 Stockholm, Sweden; 3 The Nature Conservancy, New Jersey 08314 Delmont, USA

**Keywords:** Expert judgment, Analytic Hierarchy Process, Transportation planning, Decision support, SMCA

## Abstract

Inadequate infrastructural networks can be detrimental to society if transport between locations becomes hindered or delayed, especially due to natural hazards which are difficult to control. Thus determining natural hazard susceptible areas and incorporating them in the initial planning process, may reduce infrastructural damages in the long run. The objective of this study was to evaluate the usefulness of expert judgments for assessing natural hazard susceptibility through a spatial multi-criteria analysis approach using hydrological, geological, and land use factors. To utilize spatial multi-criteria analysis for decision support, an analytic hierarchy process was adopted where expert judgments were evaluated individually and in an aggregated manner. The estimates of susceptible areas were then compared with the methods weighted linear combination using equal weights and factor interaction method. Results showed that inundation received the highest susceptibility. Using expert judgment showed to perform almost the same as equal weighting where the difference in susceptibility between the two for inundation was around 4%. The results also showed that downscaling could negatively affect the susceptibility assessment and be highly misleading. Susceptibility assessment through spatial multi-criteria analysis is useful for decision support in early road planning despite its limitation to the selection and use of decision rules and criteria. A natural hazard spatial multi-criteria analysis could be used to indicate areas where more investigations need to be undertaken from a natural hazard point of view, and to identify areas thought to have higher susceptibility along existing roads where mitigation measures could be targeted after in-situ investigations.

## Introduction

Functional infrastructures such as road and railway networks are among the key factors to a country’s economic growth. Typically, this growth is ascribed to the flow of both goods and people, locally and internationally, through transport network systems. Inadequate infrastructural networks could therefore be detrimental to a society if the transport between locations are hindered or delayed (Jenelius 2010; Oswald Beiler and Treat 2015). Logistical hindrances can often be avoided whereas natural hindrances are more difficult to control. One common natural hazard that can have an adverse effect on both infrastructure and society (people, industries, environment and organizations) is inundation by water (flooding). Flooding is a complicated hazard to consider as it results from the complex mixture of geological, geomorphological and hydrological conditions (Wu and Sidle 1995; Glade 1998; Kalantari et al. 2014b). The effect on society by natural disasters such as flooding is likely to increase due to a changed climate with increasing precipitation (IPCC 2014; MSB 2014). This is troublesome given the relatively limited understanding planners and researchers have with regards to current interactions of built-up environments and natural landscapes let alone future interactions. Therefore, there is a need for improved risk prevention and mitigation of natural hazards as their impacts on infrastructure are relevant now as well as into the future (Kalantari and Folkeson 2013).

For example, culverts and ditches are usually used to mitigate inundation risks along roads. However, if these constructions are obstructed by debris then flooding might cause damage to road infrastructure during an intense rainfall event (Kalantari et al. 2014b). The intense and heavy rainfall events can also trigger other natural hazards, e.g., landslides (Griffiths et al. 2009; Ghosh et al. 2012; Feizizadeh and Blaschke 2013) further compounding the issue. This is due to the shift between the strength of the soil and the acting stress developed under saturated conditions (Ho et al. 2012). The infrastructural disruptions caused by landslides are similar to that of inundation and increase the cost for maintenance considerably (Saha et al. 2005; Feizizadeh and Blaschke 2013). Further, the coupled interaction between culvert and ditch maintenance and potentials for natural hazards such as inundation and landslides have the ability to increase under climatic shifts (Kalantari et al. 2014a). The Swedish Transport Administration (STA), in its “Strategy for Climate Adaptation” (Liljegren 2014), has made the identification of areas prone to natural hazards one of its priorities in the road planning process. Oswald Beiler and Treat (2015) stated that applying the IPCCs definition of adaption would for transport infrastructure networks involve the evaluation of the potential risks and impacts associated with the network, as well as determining the opportunities for future sustainable development. By determining these “risk” or susceptible areas and incorporating them in the decision process, it may be possible to avoid or reduce the damage that they could cause to infrastructure (Saha et al. 2005; Ho et al. 2012; Kalantari et al. 2014b).

In order to locate areas along transport infrastructure with high natural hazard susceptibility, Geographic Information Systems (GIS) are useful since large volumes of multidisciplinary data can be handled and processed in various ways with less expenditure, i.e., time and effort (Ghosh et al. 2012; Karlsson et al. 2014). However, when a decision problem concerns road planning, a multitude of factors will influence how suitable alternatives are designed and evaluated. This implies that quantitative models are not always the deciding factors but that expert judgments also need to be considered. For such decision problems, the application of a multi-criteria analysis process can be beneficial.

Multi-criteria analysis is a part of decision analysis which provides a set of procedures for analyzing complex decision problems. By dividing the decision problem into small understandable parts, i.e., criteria, it is possible to analyze each part and combine them in a logical manner in order to produce a meaningful solution (Malczewski 1999). When combining GIS with multi-criteria analysis, i.e., spatial multi-criteria analysis (SMCA), decision makers can obtain valuable information about the consequences, uncertainties and their spatial distribution (Feizizadeh and Blaschke 2013). In many cases an SMCA is performed either to derive the initial alternatives, decide on one alternative from a set of alternatives, or to depict impacts of differing perspectives such as social, economic or environmental.

An attractive aspect with SMCA is that the scale on which these alternatives or impacts are measured is not inherently required to be a monetary scale (Meyer et al. 2009). An example of this is where the impacts are measured on a suitability scale, meaning that the results identify areas of suitability based on a grading scale of increasing impact rather than cost increase or decrease. This was for instance done by Geneletti (2005) who compared the impacts of alternative road corridors using multi-criteria analysis in combination with GIS, and Bagli et al. (2011) who identified suitable routes for power lines.

The European Union Floods Directive (Directive 2007/60/EC 2007) states that the development of flood hazard maps is essential for optimum flood management. However, for planning a sustainable road network it is of equal importance to identify other natural hazard prone areas, such as landslide and debris flow, where targeted slope-stabilization measures could be cost-beneficial. The identification of areas with high susceptibility enables the development of risk maps. SMCA have also been used for these purposes. For instance Barredo et al. (2000), Ainon et al. (2012) and Feizizadeh and Blaschke (2013) used GIS and multi-criteria analysis for landslide hazard assessment, and Fernández and Lutz (2010) combined GIS with multi-criteria analysis for urban flood hazard zoning. Peng et al. (2012) used GIS and multi-criteria analysis for natural hazard and disaster prevention. The versatility of the use of SMCA for planning is fairly high; requirements are the availability of geographic data as well as a knowledgebase for integrating scientific knowledge, including expert judgments, in the planning process. However, if expert judgments are included in a multi-criteria analysis it is important to remember that it is difficult for experts to be consistent or coherent when making judgments, especially pairwise comparison judgments (Lin and Lu 2012); and despite the widespread use of GIS and multi-criteria analysis, there is still a gap between GIS-technology, available geo-data, and accessibility for decision makers (Oswald Beiler and Treat 2015).

With this in mind, the objective of this paper was to evaluate the impact and usefulness of expert judgments in natural hazard susceptibility assessments for transport infrastructure planning from a geological data point of view. The expert judgments were aggregated into three scoring sets by skewing the scores and evaluated for three perspectives, i.e., inundation, landslide and debris flow for a study along road objects 240, 824, and 62 in Värmland County, Sweden. An SMCA approach using hydrological, geological and land use factors were implemented using two decision rules: AHP with and without aggregation, and weighted linear combination (WLC) by using equal weights and a method developed by Shaban et al. (2001), which is in this paper referred to as the factor interaction method (FIM). The resulting differences in the susceptibility assessments were identified, along with the impact of using expert judgments as well as historic incidences of natural disasters, in order to discuss the usefulness and robustness of the model as a decision support tool in an early stage of road planning.

## Materials and Methods

### Study Area

**Fig. 1:**
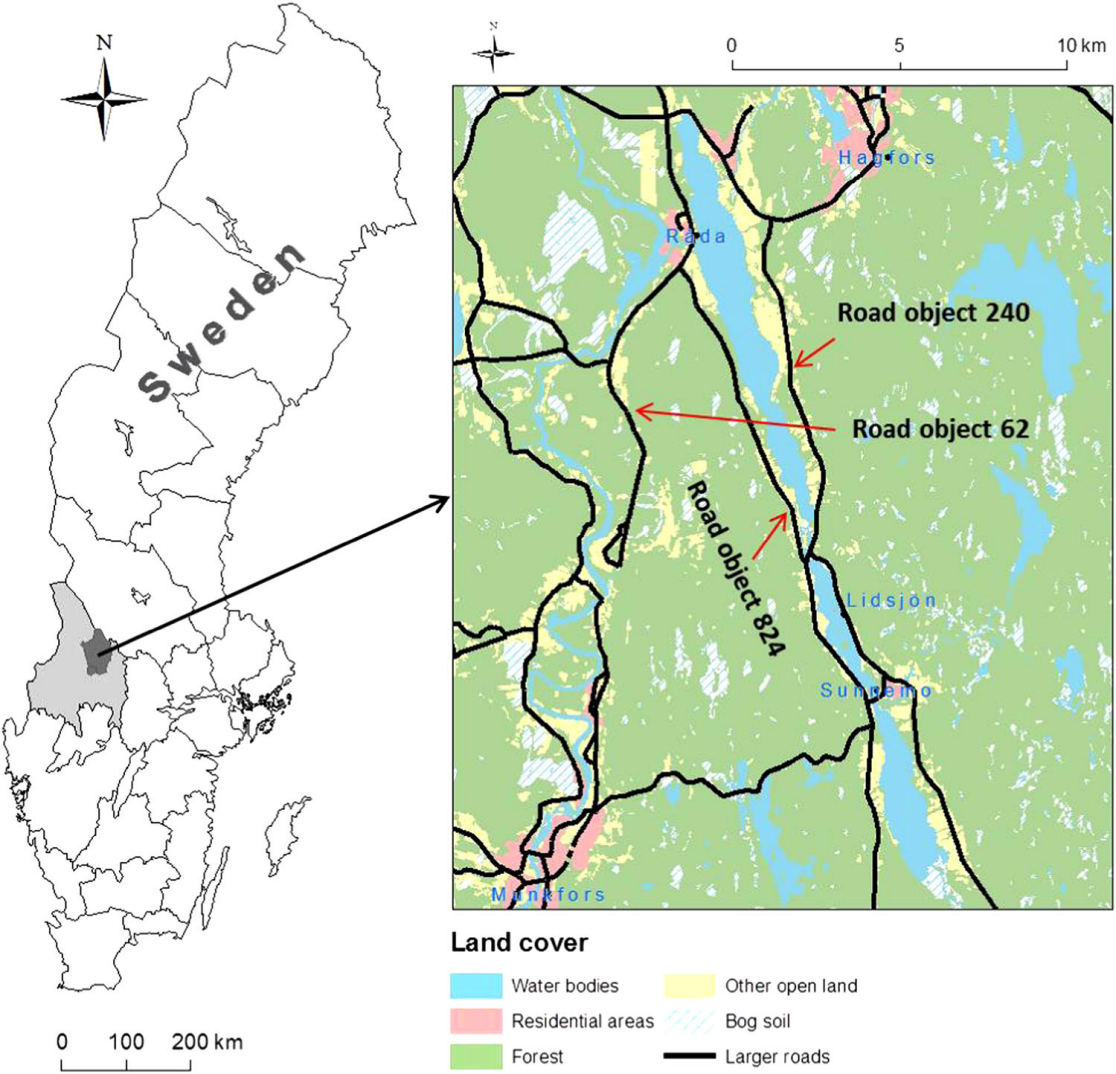
Sweden with its territorial and county borders and the location of the study area between the two towns of Hagfors and Munkfors. Spatial data © Lantmäteriet [i2012/920] (NLSS 2013), coordinate system Sweref 99 TM

**Fig. 2:**
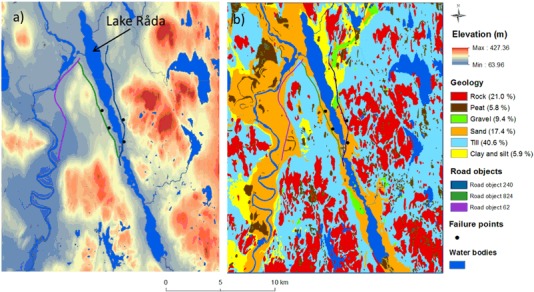
**a** Digital elevation model (DEM), and **b** Geological map of the study area. Spatial data © Lantmäteriet [i2015/920] (NLSS 2013), geological data © SGU (2013), coordinate system Sweref 99 TM

The study area is situated in the western part of Sweden, in Värmland County, between the two municipalities of Hagfors and Munkfors (Fig. 1). Minimum and maximum elevation, mean annual precipitation, and temperature are 64 m, 427 m, 775 mm (1961–2012) and, 4.5°C, respectively (NLSS 2013; SMHI 2013) (Fig. 2a). The main soil type in the two municipalities is till (54.3%) consisting of poorly-sorted quaternary unconsolidated glacial sediment with a grain-size distribution ranging from clay to boulders (Andersen and Borns 1197; Flint 1971) (Fig. 2b). There are also occurrences of glacial river sediments and sand in the study area.

**Fig. 3:**
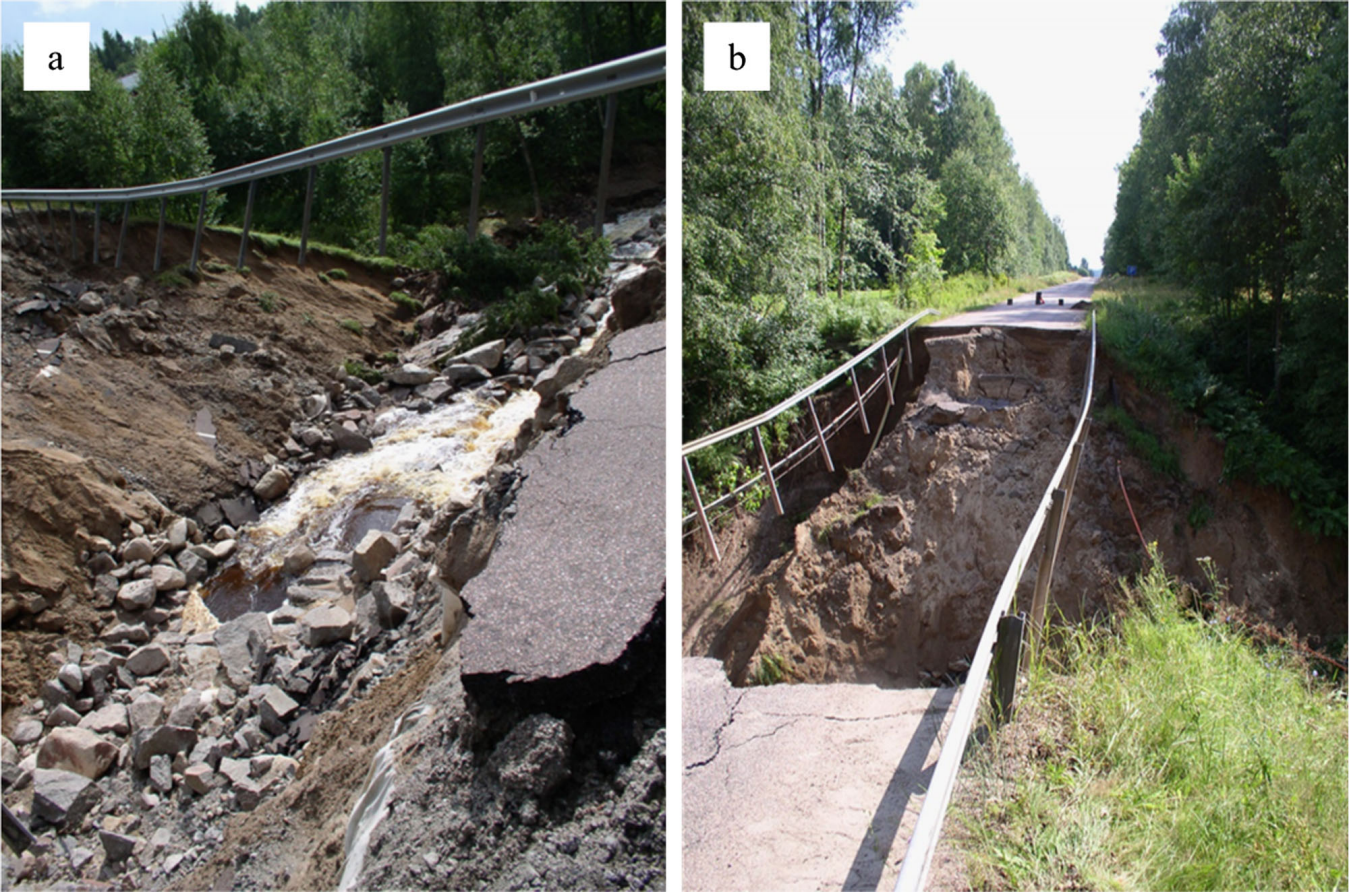
**a** and **b**: Road and embankment failures in Värmland County 2004. With permission from Thomas Morling, Swedish Transport Administration

This part of Värmland County has had several problems related to flooding of roads, especially around the three road objects 240, 824, and 62 located near Lake Råda (Fig. 1). Several road and embankment failures occurred (Fig. 3a–b) after a heavy rainfall (~200 mm in 12 h) in August 2004. This heavy rainfall resulted in large infrastructural disruptions due to high flows and extreme flooding, especially around the road objects 240 and 824. The disruptions also caused a critical situation for the residents since several roads were left either impassable or washed away (Kalantari et al. 2014b).

### Data, Criteria, and Standardization

For the SMCA two GIS software packages were used, ArcGIS 10 (ESRI 2010) and IDRISI Andes (Eastman 2006) where the latter can handle SMCA with the AHP approach. The geographic data, resolution, format and sources that were used for creating the various criteria for the SMCA are listed in Table 1. The criteria used in a SMCA can be either a factor or a constraint. Factors are criteria which either enhances or detracts from the suitability assessment for the activity under consideration. A constraint on the other hand serves to completely exclude an area from the susceptibility assessment (Eastman 2006). The selection of evaluation criteria is important since the inclusion or exclusion could have a considerable effect on the final results (Meyer et al. 2009). Although there are no universal guidelines for selecting the evaluation criteria, the criteria chosen should encompass the whole problem without increasing the complexity of the evaluation process (Meyer et al. 2009; Feizizadeh and Blaschke 2013).

**Table 1:** Data used for the SMCA modeling

Data name	Source	Horizontal resolution	Vertical resolution	Format	Description
Soil thickness data	SGU*	10 m	–	Raster	General distribution of soil thickness based on Inverse Distance Weighted (IDW) interpolation
Geological data	SGU*	1: 50,000	–	Polygon feature	Distribution of soils and rock outcrops in or near the ground surface as well as the occurrence of blocks. The soils are classified based on formation and grain size composition
DEM	NLSS**	2 m	<0.5 m	Raster	The original 2 m DEM from NLSS was resampled to 5 m to reduce noise
Land cover	NLSS**	25 m		Raster	Data identifying the distribution of vegetation and other land cover
Property data	NLSS**		****	Polygon features	Property data containing information about buildings, roads and property boundaries etc
Validation points	STA***	–	–	Point data	Point data identifying the road and embankment failures along road object 240, 824 and 62

*Geological Survey of Sweden (2012)

**National Land Survey of Sweden (2012)

***Swedish Transport Administration (2012)

****Appropriate for visualization in scale between 1:5000–1:20,000

Kalantari et al. (2014b) found that the flood probability in the study area was related to many factors, such as topography, soil texture and land use. Based on the significance of the topographic wetness index (TWI), road density, local channel slope, soil properties and land use in their prediction model, they concluded that these factors were likely strong indicators for prediction of flood probability. Kourgialas and Karatzas (2011) considered flow accumulation, slope, land use, rainfall intensity, geology and elevation in their flood hazard assessment study. These factors as well as properties such as geology (i.e., soil type and rock outcrops), soil properties, aspect, curvature, elevation, and distance to water bodies are also important to consider for both landslide (Lepore et al. 2012; Feizizadeh and Blaschke 2013) and debris flow susceptibility assessments (Jakob and Hungr 2005). Even though there are no universal guidelines on selection of criteria, Saaty and Ozdemir (2003) demonstrated that in making preference judgments as in AHP the number of elements in the group should not be more than seven. The reason for this is founded in the consistency of information derived from relations among the elements. As the number of elements increases past seven, the increase in inconsistency becomes too small for the mind to single out the element which causes the greatest inconsistency, see for instance Miller (1956).

Therefore, for the current SMCA study seven criteria, i.e., land cover, geology, soil thickness, slope angle, TWI, distance to streams and distance to lakes, were selected based on previous studies, as well as the geological and topographical characteristics of the study area (Saaty and Ozdemir 2003; Saha et al. 2005; Kourgialas and Karatzas 2011; Kalantari et al. 2014b; Lepore et al. 2012; Feizizadeh and Blaschke 2013). The chosen criteria were all also found to be important for the three perspectives inundation, landslide and debris flow and functions as common denominators between the three. The criteria were also identified as important from a geological point of view. These criteria, although many existent in previous research of various SMCA objectives (e.g., Anagnostopoulos and Vavatsikos 2012), has as far as the authors are concerned, not been addressed solely on their importance from a geological susceptibility point of view.

**Fig. 4:**
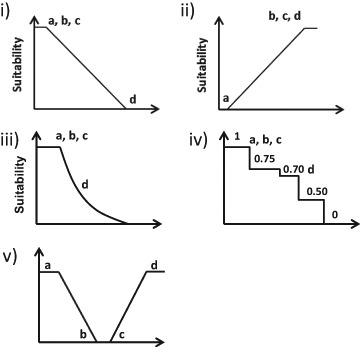
Fuzzy membership functions where: (i) linear monotonically decreasing, (ii) linear monotonically increasing, (iii) J-shaped monotonically decreasing, (iv) user defined decreasing, and (v) user defined symmetrical

These factors were then ranked and standardized in order to transform and rescale them into comparable units, scores, by using reclassification and/or fuzzy set membership functions. Fuzzy sets are classes with soft boundaries indicating that the transition between membership and non-membership of a location is gradual and ranging from 0 to 1 in value. A value close to 0 indicates a class with non-membership and a value close to 1 indicates the opposite. The difference between fuzzy sets and classical binary sets is the strictness of membership, where in classical binary sets the degree of membership is either 0 or 1, never between (Feizizadeh and Blaschke 2013). In this study fuzzy sets were used to indicate the possibility of one class belonging to another class, i.e., the overlapping between the classification values using gradual membership functions. Threshold values were defined for each membership function either by using values found in literature, expert judgment, or by using minimum and maximum values (Table 2) (Fig. 4).

**Table 2:** Fuzzy membership threshold values, based on either the interval obtained for the factor or on previously used values, i.e., see references

Factor	Fuzzy Set Membership function	Threshold value				
	M.I (Monotonically increasing)					
	M.D (Monotonically decreasing)					
	Inundation	Land sliding	Debris flow	Inundation	Land sliding	Debris flow
TWI	Linear M.D	Linear M.D	Linear M.D	*c* = 2; *d *= 37	*c* =2; *d* =37	*c* = 2; *d *= 37
	(Fig. 4i)	(Fig. 4i)	(Fig. 4i)			
Land cover	User defined	J-shaped M.D	J-shaped M.D	*c* = 1; *d *=3	*c* = 2; *d *= 4	*c* = 2; *d* = 4
	(Fig. 4iv)	(Fig. 4iii)	(Fig. 4iii)			
Geology	J-shaped M.D	J-Shaped M.D	J-shaped M.D	*c* = 2; *d* = 4	*c* = 2; *d *= 4	*c *= 2; *d* = 4
	(Fig. 4iii)	(Fig. 4iii)	(Fig. 4iii)			
Soil thickness	Linear M.I	Linear M.D	Linear M.D	*a *=0 m; *b* =103 m	*c* = 0 m; *d* =103 m	*c *= 0 m; *d* =103 m
	(Fig. 4ii)	(Fig. 4ii)	(Fig. 4ii)			
Slope angle	Linear M.I(Fig. 4ii)	User defined(Fig. 4v)	User defined(Fig. 4v)	*a* = 0°; *b* = 4° (Villa and Gianinetto 2006)	*a* = 5°*; *b* = 10°**; *c* = 44°***; *d* = 50°	*a* =10°; *b * = 20°; *c* = 45°; *d *= 55°
					* (MSB 2014)	(Jakob and Hungr 2005)
					** (Caragounis 2014)	
					*** (Coe et al. 2004)	
Distance to streams	Linear M.I(Fig. 4ii)	Linear M.I(Fig. 4ii)	Linear M.I(Fig. 4ii)	*a* =0 m; *b* = 200 m (Barredo et al. 2000)	*a* =0 m; *b*=300 m (Che et al. 2012)	*a *=0 m; *b* =200 m (Barredo et al. 2000)
Distance to lakes	Linear M.I(Fig. 4ii)	Linear M.I(Fig. 4ii)	Linear M.I(Fig. 4ii)	*a* = 0 m; *b* = 200 m (Barredo et al. 2000)	*a *=0 m; *b *=300 m (Che et al. 2012)	*a* =0 m; *b* =200 m (Barredo et al. 2000)

### Factors Considered

In the following, a brief description of the source data and processing associated with the seven selected factors, i.e., land cover, geology, soil thickness, slope angle, distance to streams and distance to lakes, is presented. While not exhaustive, these factors provide a good cross section of geologically relevant control variables that potentially affect natural hazards in the region of the study area.

The *land cover* factor contained information about the land cover type in the area. For this study five classes (Table 3) were chosen for all three perspectives based on the classes used in Kalantari et al. (2014b). These classes were reclassified for each perspective to an ordinal scale based on the probability of each land cover type’s contribution to the particular natural hazard investigated (Table 3). A lower value indicated a lower contribution, thus a lower susceptibility (i.e., high suitability for proper placement for transport infrastructure construction) and vice versa.

**Table 3:** Criteria for the perspectives and the ranking of the two criteria Land cover and Geology into suitability classes, where high susceptibility corresponds to low suitability for road construction and vice versa

Factor	Land cover	Geology
Perspective		
Inundation	Wetlands and water bodies (5)	Clay and silt (6)
	Urban (4)	Rock (5)
	Agriculture (3)	Peat (4)
	Grassland (2)	Till (3)
	Forest (1)	Sand (2)
		Gravel (1)
Landslide	Urban (5)	Clay and silt (6)
	Agriculture (4)	Peat (5)
	Grassland (3)	Sand (4)
	Forest (2)	Gravel (3)
	Wetlands and water bodies (1)	Till (2)
		Rock (1)
Debris flow	Grassland (5)	Sand (6)
	Agriculture (4)	Gravel (5)
	Forest (3)	Till (4)
	Wetlands and water bodies (2)	Peat (3)
	Urban (1)	Clay and silt (2)
		Rock (1)

**Fig. 5:**
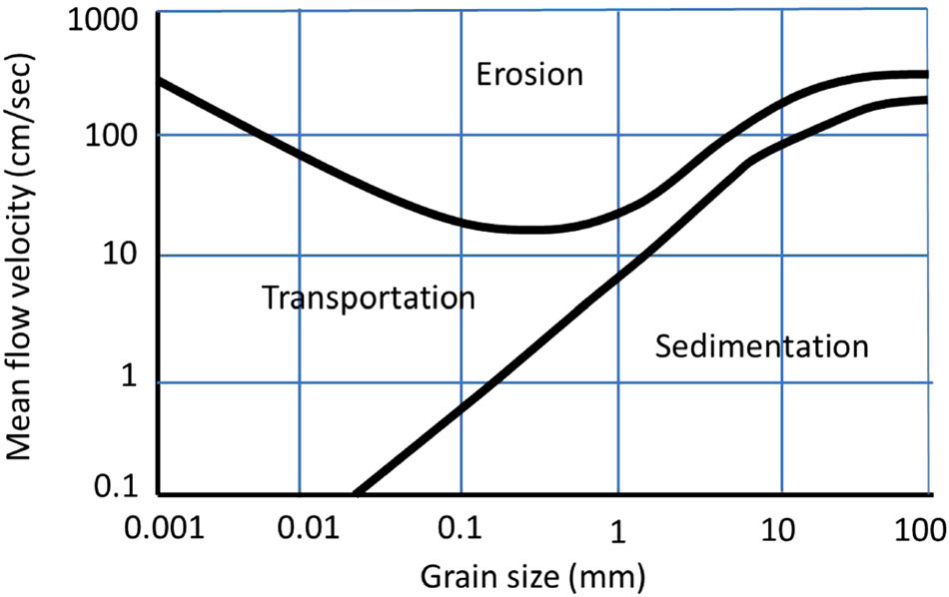
The Hjulströms diagram used for criteria classes in the perspective of debris flow. First appeared in Hjulström (1935)

The *geological* factor encompassing both soil types and rock outcrops was used to indicate the distribution of the six selected soil types at ground surface (Table 3); based on the variable classes in Kalantari et al. (2014b). The reclassification for each perspective to an ordinal scale was undertaken similarly to the procedure for land cover, i.e., a low value indicated a low contribution to susceptibility and thus high suitability for road construction. For ranking the suitability classes of the factors for the perspective debris flow, the Hjulströms diagram (Fig. 5) was used as a guideline. Hydrologists and geologists use this diagram to determine whether a stream flow erodes, transports, or deposits sediments depending on the fluid velocity and the size of the sediment (Crespin et al. 2014). In this case the overland flow on the land surface was considered to act as a temporary shallow river, and the same principles behind the diagram were assumed applicable.

The *soil thickness* factor was used to indicate the depth of the soil layer from the ground surface until the surface of the basement rock is reached. The soil thickness factor was based on an inverse distance weighted interpolation model using point data with soil thickness information (Karlsson et al. 2014) developed by SGU (Daniels and Thunholm 2014) and used in transport infrastructure planning studies (e.g., Karlson et al. 2016). Since the soil thickness factor was initially in a continuous scale this factor was not reclassified into several individual suitability classes. Instead the soil thickness contribution to the particular investigated perspective was based on the gradual increase in thickness; where either a shallow thickness would be less susceptible or more susceptible depending on perspective (Table 2). The same was applied for the following factors due to their initial continuous scale: *Slope angle*, *TWI*, *Distance to streams*, and *Distance to lakes*.

The *slope angle* factor identified the slope or rate of maximum change in elevation. It was calculated by using the digital elevation model (DEM) and measured in degrees. The *TWI* factor was chosen because of its representation of the water accumulation in a catchment (Sørensen et al. 2006) and is defined as (Eq. 1):

1 $$TWI = {\rm{ln}}\frac{a}{{\tan \beta }}$$

where *α* is the local upslope area draining through a certain point per unit contour length and *Tan β* is the local slope. However, to be able to use Eq. 1 in a GIS environment it was modified (Eq. 2):

2 $$\begin{array}{ccccc}\\ TWI & = \ln \displaystyle \frac{{upslope\,contributing\,area}}{{\tan \left( {slope\,angle} \right)}}= \ln \displaystyle \frac{{As}}{{\tan \left( \alpha \right)}}\\ \\ & \,\, = \ln \displaystyle \frac{{\left( {Flow\,accumulation + 1} \right) \times {{\left( {Pixel\,size} \right)}^2}}}{{\tan \left( \alpha \right)}}\\ \end{array}$$

where *As* is the up-slope contributing area per unit contour width, but since the resolution, i.e., pixel size, can be considered as a proxy for the contour width it was used instead. This is a fairly common approximation made for estimating TWI values in catchments.

The *distance to streams* and *distance to lakes* factors was created by using the ‘Euclidean distance’ in ArcGIS. This tool calculated the distance in meters from each stream or each lake to the end of the study area.

### The SMCA Approach

**Fig. 6:**
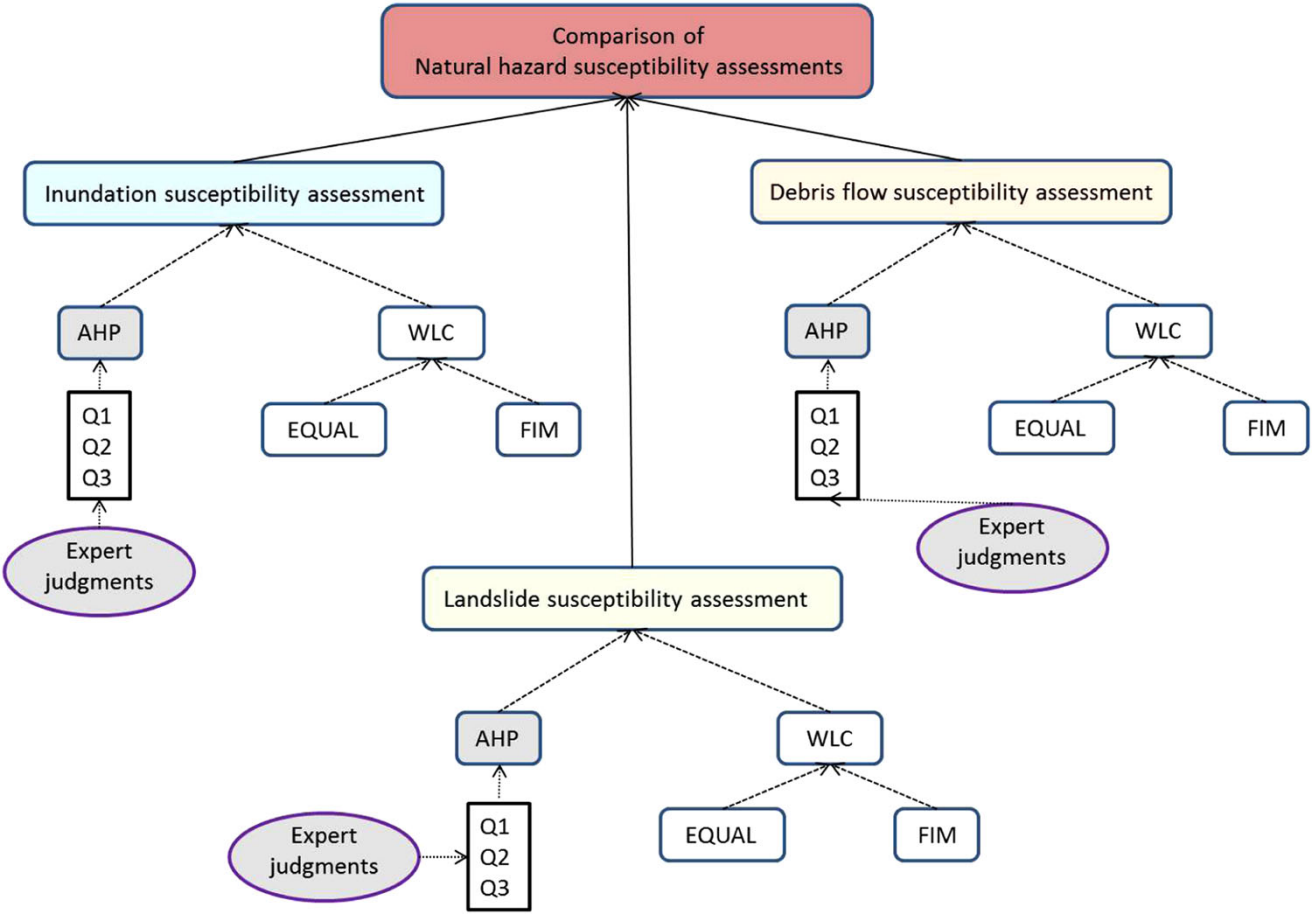
Illustration of the conceptual model

In order to find susceptible areas for natural hazards in view of road localization, three perspectives of susceptibility were chosen for the SMCA: inundation (Johnson and Warburton 2002; Wu and Sidle 1995), landslide (Glade 1998), and debris flow (Jakob and Hungr 2005) (Fig. 6). These were based on the various negative impacts these can have on the road and railway structures. Direct or indirect methods can be used for the susceptibility mapping. When performing a direct mapping method the susceptible regions are identified by comparing geographical data with known occurrences at other locations (Feizizadeh and Blaschke 2013). The indirect mapping method on the other hand integrates factors and weighs their importance using more or less subjective decision-making rules, based on expert judgment (Feizizadeh and Blaschke 2013). Since known occurrences are few, as often in risk assessment, the latter mapping method was used for this susceptibility study.

For SMCA it is important to decide what decision rule to use, since it defines the way the criteria are aggregated to an overall assessment under the influence of the weights given to each of them (Meyer et al. 2009). For the susceptibility assessment two decision rules (AHP and WLC) were selected and compared (Fig. 6). The AHP is a multi-attribute weighting method (Malczewski 1999; Feizizadeh and Blaschke 2013) where ratio scales can be derived from paired comparisons using the Saaty scale of intensity of importance (Table 4) (Saaty 2008a). AHP allows the use of both quantitative and qualitative information (Feizizadeh and Blaschke 2013) and is based on four steps: i) problem definition, ii) structuring the hierarchy tree, iii) comparative judgment, and iv) synthesis of priorities (Malczewski 1999; Saaty 2008a).

**Table 4:** The Saaty scale of intensity of importance

Continuous Rating Scale (Intensity of importance)								
1/9	1/7	1/5	1/3	1	3	5	7	9
extremely	very strongly	strongly	moderately	equally	moderately	strongly	very strongly	extremely
Less important					More important			

The first step (problem definition) is critical since an unclear problem definition could lead to either too many or too few criteria. The second step is to break down the problem into a hierarchy which captures the basic and important elements of the problem. Once the hierarchy is determined the criteria should be compared pairwise and finally the priorities obtained from the comparison are used to weight the priorities (Malczewski 1999; Saaty 2008a). AHP also enables a consistency evaluation of the judgments (Feizizadeh and Blaschke 2013) by calculating a consistency ratio (CR) based on the weights obtained. The second decision rule selected for the susceptibility assessment was the WLC with two different weighting schemes, i.e., *equal weighting* and weighting through FIM (Shaban et al. 2001; Jamali et al. 2014). WLC is one of the most common decision rules, and is often applied in suitability analysis due to the easy implementation within a GIS environment (Malczewski 2000). Because of this widespread use and easy implementation WLC might be the decision rule planners and decision makers chose for susceptibility assessments. Therefore it was selected as the decision rule for comparison of AHP. The different steps implemented are similar to that of AHP except for the step concerning pairwise comparison of the criteria used for weighting.

**Fig. 7:**
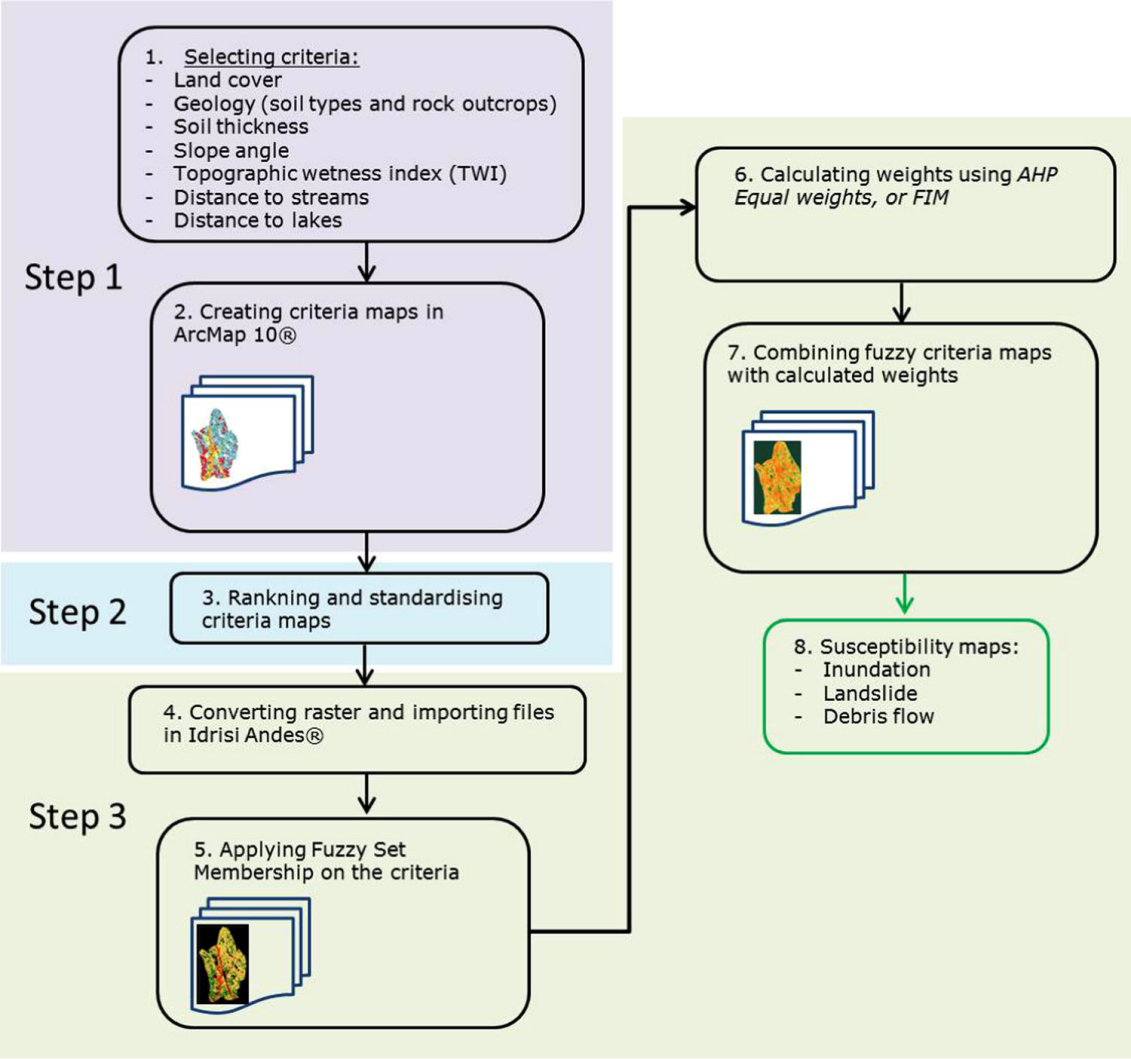
The processing steps for AHP

In order to locate areas that are considered susceptible to inundation, landslide and debris flow, a procedure consisting of three steps, for each perspective, was undertaken (Fig. 7). Firstly, the seven mentioned criteria were selected and criteria maps were prepared in ArcMap 10 (Fig. 7, Step 1). Secondly, criteria maps were created by ranking and standardizing criteria classes and the criteria themselves, which implied that values of priority were given to the selected criteria classes (Table 3) (Fig. 7, Step 2). Thirdly, the criteria maps were imported to IDRISI Andes where the decision support wizard was applied. This decision wizard was used to apply standard or user defined fuzzy membership functions to the criteria (Fig. 4), weight calculation and assignment as well as the combination of criteria and weights into susceptibility maps (Fig. 7, Step 3), which was later used for the calculation of percentage of susceptibility. The susceptibility was outlined for all three perspectives; considering that areas with higher susceptibility had lower suitability and vice versa.

### Weighting

Weighting was used to indicate the judgment of importance of the factors, and the values selected have a major impact on the outcome (Meyer et al. 2009). Three weighting schemes were used in the susceptibility assessment for the three perspectives.

#### Weighting for AHP

The weighting values were calculated based on pairwise comparison scores given by different expert groups according to the Saaty scale (Table 4). Three pairwise comparison matrices, one for each perspective, were sent to 20 experts from academia, STA, SGU, and the Swedish Meteorological and Hydrological Institute (SMHI) with a request to rank the factors using the Saaty scale according to their perception of their relative importance. The objective was to select experts from different knowledge backgrounds which contribute to the entire road and railway infrastructure processes, i.e., from planning to maintenance. Responses were received from eight experts including those who responded after a reminder. These answers came from experts in academia as either professors or professor emeritus in environmental hydrology, environmental geology and land and water resources sciences. The other experts had backgrounds as department head and/or consultant for roads and railway, geoengineering, infrastructure investment and EIA for more than 10 years. The first quartile Q1 (25th percentile), average Q2 (50th percentile), and third quartile Q3 (75th percentile) scores from the experts were calculated and included into nine separate matrices (one set encompassing three matrices for each perspective) (Tables 5–7) that would represent the comparison scores and later used to calculate the factor weights (Table 8).

**Table 5:** Pairwise comparison matrix for the three perspectives Q1

Aggregated scores Q1 (25th percentile)							
Inundation	Slope	Geology	Land cover	Soil thickness	Distance to streams	Distance to lakes	TWI
Slope							
Geology	1						
Land cover	0.4	1					
Soil thickness	0.22	1	1.20				
Distance to streams	3.45	3.45	5.55	7.14			
Distance to lakes	3.33	3.33	5	5.26	1		
TWI	3.03	3.03	3.03	5.26	3.03	2	

Landslide	Slope	Geology	Land cover	Soil thickness	Distance to streams	Distance to lakes	TWI
Slope							
Geology	1.20						
Land cover	0.4	0.33					
Soil thickness	0.4	0.4	3.03				
Distance to streams	1	0.4	3.03	1			
Distance to lakes	0.4	0.25	1.20	1	1		
TWI	0.33	1	3.03	1	3.03	5	

Debris flow	Slope	Geology	Land cover	Soil thickness	Distance to streams	Distance to lakes	TWI
Slope							
Geology	1						
Land cover	0.40	1					
Soil thickness	0.40	1	3.03				
Distance to streams	0.40	0.25	1.14	1			
Distance to lakes	0.40	0.20	1.14	1	1		
TWI	1	2.00	3.03	7.14	7.14	7.14

**Table 6:** Pairwise comparison matrix for the three perspectives Q2

Aggregated scores Q2 (50th percentile)							
Inundation	Slope	Geology	Land cover	Soil thickness	Distance to streams	Distance to lakes	TWI
Slope							
Geology	0.38						
Land cover	0.26	0.32					
Soil thickness	0.19	0.46	0.28				
Distance to streams	0.47	0.47	0.63	0.93			
Distance to lakes	0.42	0.47	0.62	0.63	0.52		
TWI	0.61	1.92	1.92	2.04	0.51	0.48	

Landslide	Slope	Geology	Land cover	Soil thickness	Distance to streams	Distance to lakes	TWI
Slope							
Geology	0.41						
Land cover	0.21	0.20					
Soil thickness	0.36	0.29	0.31				
Distance to streams	0.19	0.22	0.36	0.41			
Distance to lakes	0.18	0.19	0.39	0.41	0.72		
TWI	0.40	0.41	0.73	0.66	1.04	1.09	

Debris flow	Slope	Geology	Land cover	Soil thickness	Distance to streams	Distance to lakes	TWI
Slope							
Geology	0.31						
Land cover	0.27	0.29					
Soil thickness	0.29	0.31	0.25				
Distance to streams	0.29	0.21	0.28	0.40			
Distance to lakes	0.22	0.17	0.23	0.28	0.40		
TWI	0.46	0.93	0.77	0.81	1.27	2.17

**Table 7:** Pairwise comparison matrix for the three perspectives Q3

Aggregated scores Q3 (75th percentile)							
Inundation	Slope	Geology	Land cover	Soil thickness	Distance to streams	Distance to lakes	TWI
Slope							
Geology	0.33						
Land cover	0.2	0.29					
Soil thickness	0.14	0.33	0.14				
Distance to streams	0.5	0.5	1	1			
Distance to lakes	0.29	0.5	1	1	0.33		
TWI	0.4	1.20	1.20	1.20	0.4	0.4	

Landslide	Slope	Geology	Land cover	Soil thickness	Distance to streams	Distance to lakes	TWI
Slope							
Geology	0.36						
Land cover	0.14	0.14					
Soil thickness	0.33	0.25	0.18				
Distance to streams	0.13	0.18	0.29	0.29			
Distance to lakes	0.13	0.13	0.29	0.2	0.8		
TWI	0.33	0.33	1	1	1	1	

Debris flow	Slope	Geology	Land cover	Soil thickness	Distance to streams	Distance to lakes	TWI
Slope							
Geology	0.20						
Land cover	0.18	0.20					
Soil thickness	0.20	0.20	0.14				
Distance to streams	0.20	0.18	0.18	0.29			
Distance to lakes	0.14	0.13	0.13	0.18	0.33		
TWI	0.33	1	1	1	1.20	1.20

**Table 8:** Weighting scheme for AHP

Weighting scheme (%)								
AHP individual experts								
Inundation	Exp. 1	Exp. 2	Exp. 3	Exp.4	Exp. 5	Exp.6	Exp. 7	Exp. 8
Slope	0.0721	0.1573	0.1847	0.1821	0.2600	0.1461	0.4555	0.1138
Geology	0.0296	0.1030	0.2208	0.4394	0.1005	0.0607	0.0857	0.0409
Land cover	0.0296	0.1529	0.0924	0.1642	0.0452	0.1072	0.0941	0.0286
Soil thickness	0.0278	0.0254	0.1144	0.0897	0.0377	0.0494	0.0713	0.0526
Distance to streams	0.4905	0.2082	0.1144	0.0626	0.3325	0.0453	0.1053	0.2447
Distance to lakes	0.2648	0.2082	0.0872	0.0620	0.2242	0.0453	0.1053	0.2601
TWI	0.0856	0.1451	0.1865	n/a	n/a	0.5459	0.0829	0.2594

Landslide	Exp. 1	Exp. 2	Exp. 3	Exp.4	Exp. 5	Exp.6	Exp. 7	Exp. 8
Slope	0.2307	0.1799	0.1847	0.2072	0.3689	0.1461	0.4668	0.3871
Geology	0.4344	0.2071	0.2208	0.3866	0.3005	0.0607	0.1792	0.2016
Land cover	0.0987	0.0566	0.0924	0.1813	0.0362	0.1072	0.1594	0.0643
Soil thickness	0.0434	0.0926	0.1144	0.0797	0.1932	0.0494	0.0636	0.1799
Distance to streams	0.0578	0.1699	0.1140	0.0726	0.0652	0.0453	0.0355	0.0358
Distance to lakes	0.0470	0.1699	0.0872	0.0726	0.0360	0.0453	0.0408	0.0307
TWI	0.0880	0.1243	0.1865	n/a	n/a	0.5459	0.0548	0.1007

Debris flow	Exp. 1	Exp. 2	Exp. 3	Exp.4	Exp. 5	Exp.6	Exp. 7	Exp. 8
Slope	0.2129	0.2999	0.3320	0.1502	0.3274	0.0640	0.4204	0.4118
Geology	0.2129	0.1359	0.1770	0.3120	0.3338	0.0640	0.1696	0.1909
Land cover	0.1872	0.2319	0.0448	0.1961	0.0486	0.1170	0.0781	0.1086
Soil thickness	0.0525	0.0683	0.1016	0.1340	0.1351	0.0504	0.0461	0.1804
Distance to streams	0.0276	0.1254	0.0942	0.1043	0.0758	0.0640	0.0456	0.0428
Distance to lakes	0.0276	0.0200	0.0764	0.1035	0.0794	0.0640	0.0456	0.0262
TWI	0.2791	0.1186	0.1740	n/a	n/a	0.5764	0.1947	0.0394

AHP aggregated			
Inundation	Q1	Q2	Q3
Slope	0.0955	0.2866	0.3336
Geology	0.0650	0.1676	0.1735
Land cover	0.0501	0.1165	0.1193
Soil thickness	0.0429	0.0740	0.0595
Distance to streams	0.2253	0.1178	0.1325
Distance to lakes	0.2151	0.0968	0.0901
TWI	0.3062	0.1408	0.0915

Landslide	Q1	Q2	Q3
Slope	0.2272	0.3412	0.3740
Geology	0.2262	0.2677	0.2823
Land cover	0.0555	0.1221	0.1213
Soil thickness	0.1112	0.0920	0.0820
Distance to streams	0.1119	0.0529	0.0379
Distance to lakes	0.0729	0.0470	0.0333
TWI	0.1955	0.0771	0.0691

Debris flow	Q1	Q2	Q3
Slope	0.1896	0.3288	0.3953
Geology	0.1649	0.2274	0.2266
Land cover	0.0751	0.1524	0.1514
Soil thickness	0.0969	0.0909	0.0699
Distance to streams	0.0620	0.0589	0.0425
Distance to lakes	0.0609	0.0363	0.0269
TWI	0.3506	0.1053	0.0874

The *CR* was calculated in the IDRISI Andes decision wizard and used to measure how consistent the judgments were compared to random judgments. Firstly, the weighted sum vector was determined by multiplying the criteria weights with their respective column in the original pairwise comparison matrix. After this the calculated values were summed over the rows. The consistency vector was determined by dividing the weighted sum vector by the previously determined criterion weights (Malczewski 1999). Secondly, the consistency index was calculated,

3 $$CI = \frac{{\lambda - n}}{{n - 1}}$$

where *λ* is the average value of the consistency vector and *n* is the number of criteria being considered. Thirdly, the consistency ratio was determined,

4 $$CR = \frac{{CI}}{{RI}}$$

where *RI* is the random index (consistency index of a randomly generated pairwise comparisons matrix) dependent on the number of elements being compared (Malczewski 1999). A *CR* value less than 0.10 indicates a reasonable level of consistency in the pairwise comparisons. If the *CR* value is higher or equal to 0.10 the values in the original pairwise matrix preferably should be revised since it indicates inconsistent judgments (Malczewski 1999).

#### Weighting using WLC

**Fig. 8:**
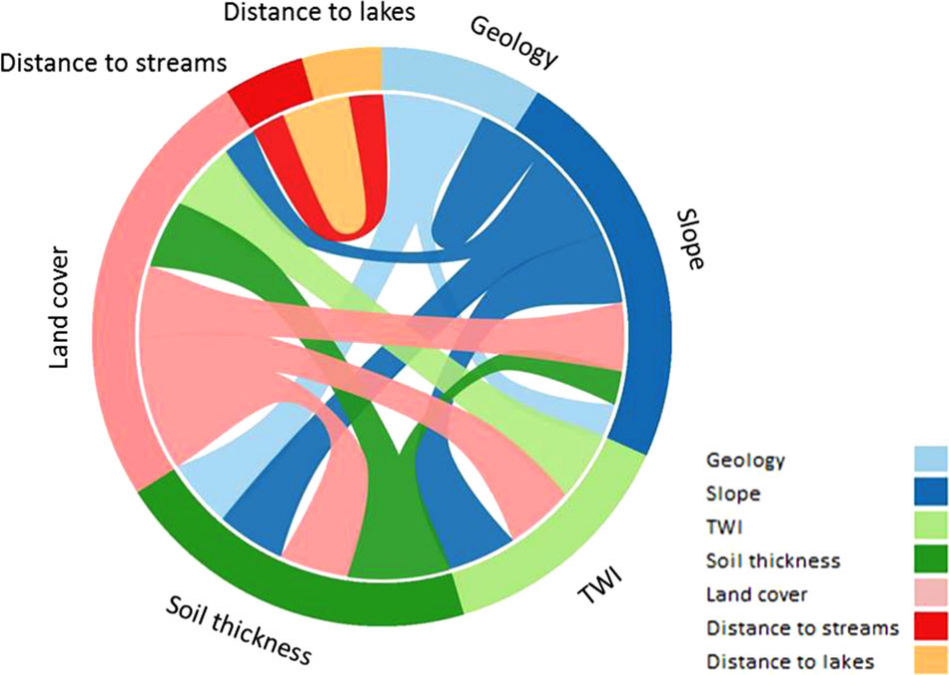
Illustration of the factor interaction method (FIM)

Two weighting schemes were used for each perspective (Table 9). The first weighting scheme was simple *equal weighting*. This weighting scheme for *equal weighting* implied that each of the seven criteria was equally important and the weight for each criterion was calculated as 100/7 (~14.3%) (Table 9). Shaban et al. (2001) presented a methodology, i.e., a weighting scheme, which considered the effect of each factor on all other factors. This weighting scheme is in this paper and in Jamali et al. (2014) referred to as FIM. The idea behind FIM is that a hazardous area cannot be estimated by considering the effect of each factor on its own; and even though an integration of all factors is necessary the factors do not have the same degree of influence (Kourgialas and Karatzas 2011). Therefore, the influence between the factors was divided into two groups, i.e., major and minor. Factors with a major influence indicate factors where a change would have a direct effect on another factor. Factors with minor influence are on the other hand considered to have a secondary effect, i.e., a change in one factor would have an indirect effect on the other (Kourgialas and Karatzas 2011; Jamali et al. 2014). The influence of each factor on the other factors is illustrated in Fig. 8. For each major effect a factor was considered to have on another factor a score of 10 points was given to it, for minor effects a score of 5 points was given, similarly to the quantification seen in Shaban et al. (2001), Kourgialas and Karatzas (2011) and Jamali et al. (2014). The weights were then calculated based on the number of points for each factor divided by the sum of points. Even though there is a potential risk of circularity between the factors in FIM the influence of this is reduced since the effect of the factors are divided into groups of major and minor effects. In this study Slope had for instance two major and two minor effects (scoring 30) (Fig. 8). TWI had one major effect (scoring 10) and Soil thickness had one major and one minor effect (scoring 15). Both Distance to streams and Distance to lakes had only one minor effect therefore scoring five each. Land cover had three major effects (scoring 30) and Geology had one major and one minor (scoring 15). Since the sum of total scores used for FIM is 110, weights were calculated by dividing scoring with the sum of total scoring, e.g., Slope for instance was given a weight of 30/110% (~27%). Once the weights were calculated the susceptibility for each perspective using WLC was obtained through the following equation:

5 $${A_i} = \mathop {\sum }\limits_j {w_j}{x_{ij}}$$

where *A*_*i*_ is the final susceptibility score in each pixel, *x*_*ij*_ is the suitability of the *i*th cell with respect to the *j*th layer, and *w*_*i*_ is the normalized weight so that$\mathop {\sum }\limits {w_i} = 1$.

**Table 9:** Weighting scheme for WLC

Weighting scheme for Weighted linear combination (WLC) in %		
	Equal weighting	Factor interaction method (FIM)
Slope	14.29	27.27
TWI	14.29	9.09
Soil thickness	14.29	13.63
Distance to streams	14.29	4.54
Distance to lakes	14.29	4.54
Land cover	14.29	27.27
Geology	14.29	13.63

### Comparison of the Susceptibility Results

A comparison of the expert scoring in AHP was undertaken. The results of the comparison explained how much the assessment of the SMCA study was influenced by the assessor judgments, which was reflected by the weights considered (Saltelli et al. 1999). In this study the comparison was undertaken by aggregating and dividing the expert scoring into the three weighting sets AHP Q1, Q2, and Q3 (Tables 5–7). The aggregated results might not be interpretable or of an acceptable consistency if the individual comparison matrices are not sufficiently consistent from the beginning (Lin and Lu 2012). However, the main objective of the aggregation procedure selected in this study was to analyze how the effect of merging the different expert scores, using the aggregation methods mentioned above, into one scoring matrix would affect the overall susceptibility assessment using AHP. Lastly compare the results from the AHP procedure using the expert judgments to the results from the susceptibility assessments using the other SMCA procedures.

In order to compare and evaluate the susceptibility for the three perspectives, regardless of SMCA procedure, the results from the SMCA was divided into two groups using a natural breaks (Jenks) classification, similarly to Oswald Beiler and Treat (2015). By using the natural breaks classification the data were divided into two classes of lower and higher susceptibility (*Class 1, low*; and *Class 2, high*) by finding natural groupings inherent in the data (ESRI 2007). The classification works by finding the break points that best would group similar values together and at the same time maximizes the differences between the classes (Appendix A). By classifying according to the natural breaks for inundation, landslide and debris flow the percentage of susceptibility scores in *Class 1* and *Class 2* could be estimated.

## Results and Discussion

**Fig. 9:**
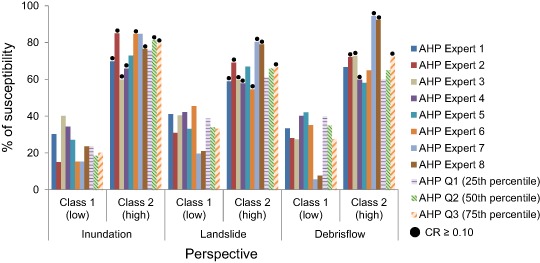
Susceptibility using individual AHP and aggregated AHP (Q1, Q2, and Q3)

**Fig. 10:**
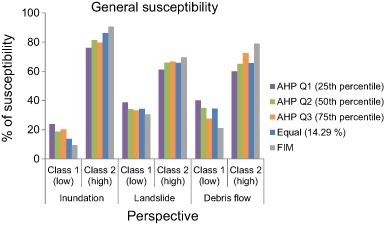
Susceptibility for the three decision rules, i.e., aggregated AHP, equal weighting, and FIM

The percentage of susceptibility scores in *Class 1* and *Class 2* differed between the individual experts and aggregated AHP susceptibility (Fig. 9, Fig. 10 and Table 10). The perspective that received the highest *Class 2* percentage for five out of eight experts was inundation (between 65.8 and 85%), followed by debris flow and landslide. When aggregating the individual expert scores (Appendix B) into the three scoring sets, AHP Q1, Q2, and Q3 (Fig. 10) (Tables 5–7), inundation received the highest *Class 2* percentage as well (76.1 to 81.3%). Inundation was then followed by landslide and debris flow.

**Table 10:** Percentage of susceptibility scores in Class 1 and Class 2 for the three perspectives

Decision rule	Inundation		Landslide		Debris flow	
	General susceptibility (entire study area)					
	Class 1 (%)	Class 2 (%)	Class 1 (%)	Class 2 (%)	Class 1 (%)	Class 2 (%)
AHP Q1 (25th percentile)	23.9	76.1	38.8	61.2	40.0	60.0
AHP Q2 (50th percentile)	18.7	81.3	34.1	65.9	35.0	65.0
AHP Q3 (75th percentile)	20.3	79.7	33.3	66.7	27.6	72.4
WLC (Equal weighting)	13.8	86.2	34.2	65.8	34.4	65.6
WLC (FIM)	9.5	90.5	30.5	69.5	21.0	79.0
Susceptibility along road object 240						
AHP Q1 (25th percentile)	18.3	81.7	56.1	43.9	52.4	47.6
AHP Q2 (50th percentile)	12.8	87.2	51.3	48.7	53.9	46.1
AHP Q3 (75th percentile)	13.9	86.1	49.2	50.8	44.5	55.5
WLC (Equal weighting)	7.2	92.8	55.4	44.6	45.7	54.3
WLC (FIM)	6.4	93.6	51.2	48.8	39.2	60.8
Susceptibility along road object 824						
AHP Q1 (25th percentile)	5.0	95.0	51.7	48.3	78.1	21.9
AHP Q2 (50th percentile)	2.2	97.8	51.2	48.8	75.0	25.0
AHP Q3 (75th percentile)	3.5	96.5	50.0	50.0	74.6	25.4
WLC (Equal weighting)	0.5	99.5	50.3	49.7	54.4	45.6
WLC (FIM)	0.7	99.3	49.7	50.3	47.9	52.1
Susceptibility along road object 62						
AHP Q1 (25th percentile)	13.6	86.4	45.4	54.6	88.2	11.8
AHP Q2 (50th percentile)	15.4	84.6	39.5	60.5	85.8	14.2
AHP Q3 (75th percentile)	20.3	79.7	36.5	63.5	82.4	17.6
WLC (Equal weighting)	2.3	97.7	45.9	54.1	75.9	24.1
WLC (FIM)	5.6	94.4	39.6	60.4	63.7	36.3
Susceptibility along the failure points						
AHP Q1 (25th percentile)	77.1	22.9	87.2	12.8	82.8	17.2
AHP Q2 (50th percentile)	5.1	94.9	73.4	26.6	79.5	20.5
AHP Q3 (75th percentile)	5.7	94.3	71.3	28.7	73.6	26.4
WLC (Equal weighting)	6.8	93.2	89.7	10.3	86.5	13.5
WLC (FIM)	0.3	99.7	73.4	26.6	75.1	24.9

**Fig. 11:**
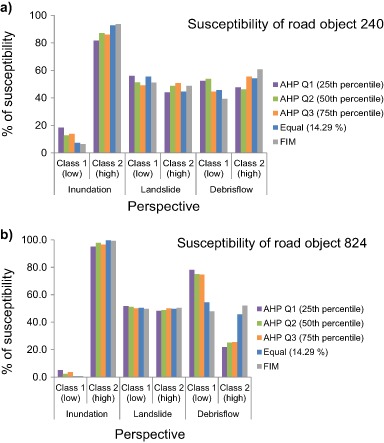
**a** Susceptibility along road object 240, and **b** susceptibility along road object 824

**Fig. 12:**
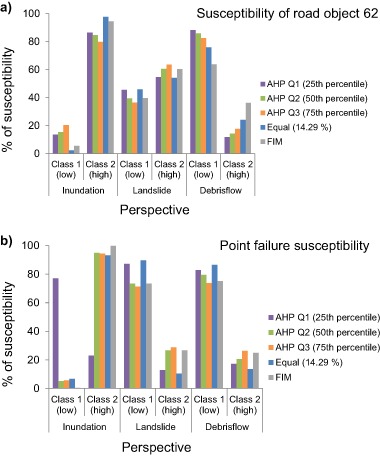
**a** Susceptibility along road object 62, and **b** susceptibility for the four failure points

In order to analyze the susceptibility for the three perspectives, downscaling was undertaken to smaller subsets of the study area, so the analysis was focused around (a) the entire study area, (b) along the three road objects close to Lake Råda and, (c) 40 m around the four failure points where road embankment failure occurred (Fig. 1). The perspective that received the highest percentage of *Class 2* susceptibility regardless of decision rule and weighting scheme was inundation (Fig. 9 and Table 10). WLC with FIM resulted in a susceptibility of 90.5%, followed by *equal weighting* and *AHP Q2*. Inundation was followed by small differences in susceptibility between both landslide and debris flow (Table 10). The susceptibility for landslide ranged from 61.2 to 69.5%. Debris flow on the other hand ranged from 60 to 79% depending on the analyzed decision rule. The susceptibility along the three road objects 240, 824, and 62 were also highest for inundation (Fig. 11a–b, and Fig. 12a).

Of the three road objects, road object 824 had the highest susceptibility to inundation of all, ranging from 95 to 99.5% depending on decision rule (Table 10). After inundation, debris flow was the perspective that received highest susceptibility for road object 240 (between 46.1 and 60.8%) followed by the second highest susceptibility for road objects 824 and 62. When studying the susceptibility for the four failure points (Fig. 12b) it was seen that inundation was also the perspective that received the highest susceptibility of all the three analyzed perspectives. For this case the susceptibility ranged from 22.9 to 99.7% (Table 10). The susceptibility of the four failure points was then followed by landslide (between 10.3 and 28.7%) and then debris flow (between 13.5 and 26.4%).

### Differences in Susceptibility between the Decision Rules AHP and WLC

#### AHP, expert knowledge, and influences of aggregating the knowledge

For this study AHP was used as one of the decision rules for comparison. Although AHP is a widely used weighting method due to the possibility to include pairwise comparisons, it has received substantial criticism from a number of MCA specialists (Department for Communities and Local Government (DCLG) 2009). The doubts with AHP can be divided into five main concerns (DCLG 2009), (1) the pairwise comparison scale which has the potential to be internally inconsistent; (2) the link between the points on the scale and the corresponding linguistic descriptions does not have a theoretical background, (3) weights are elicited for criteria before measurement scales for criteria are set, (4) introduction of new options can change relative ranking of some of the original options (i.e., rank reversal), and (5) underlying axioms on which AHP is based on might not be sufficiently clear in order to be empirically testable. As a result from the concerns partly listed above other MCA procedures have been developed in order to retain the strengths of the AHP but also avoiding the mentioned objections. One method is for instance REMBRANDT, which instead of using the Saaty scale and eigenvector, uses a direct rating system which is on a logarithmic scale and the geometric mean to identify weights (DCLG 2009). Despite the doubts raised about AHP it has several benefits, where one benefit of AHP is that it enables a decision maker to focus their attention on the development of a formal structure. This is undertaken in order to capture the important factors which likely differentiate a good option from a poor option (DCLG 2009). Another main advantage of AHP is that it allows for pairwise comparisons of factors which imply that only two criteria have to be considered at the same time by a decision maker or expert (Malczewski 1999).

In this study, the experts that were asked to participate in the ranking of the factors were affiliated at either a governmental authority with main focus on geology, hydrology, and infrastructure planning or in academia with research fields in these topics. However, the scoring method used for the susceptibility assessment through AHP can be seen as more or less subjective, and even with similar background or knowledge the scores were distributed along the range in the Saaty scale and most experts were not consistent in their scoring, i.e., *CR* ≥ 0.10 (Fig. 9) (Appendix B). It was initially assumed that the perspective that most experts would be inconsistent with would be debris flow, since debris flow is not as common and well known a natural hazard in Sweden as for example inundation and landslide. However, it turned out to be the perspective most experts were consistent with, followed by landslide and then inundation. A reason might be that debris flow was the perspective they were asked to score the last, giving them some time to re-evaluate the previous scores for the other perspectives. It could also reflect the general lack of expertize such that all surveyed experts had about the same “basic” level of experience with debris flows while each expert had specialized experience with inundation and landslides. The susceptibility assessments from the experts also varied within each perspective. Almost half of the experts scoring resulted in much higher susceptibility than the others (Fig. 9). It also seemed that most experts from academia tended to score in such a way that the percentage of *Class 2* susceptibility would be higher than for instance the geo-engineers or planners. This could support the idea of expertize specialization consistent with background. Lin and Lu (2012) had a similar statement like in this study, and found that there may be significant differences in judgments or viewpoints of people working within the same organization, or even between organizations. They also stated that experts that have more years of experience may emphasize criteria differently than experts with less experience.

Various methods for aggregating preference in AHP were proposed in order to reconcile conflicts and differences among decision makers (Lin and Lu 2012). For instance, Basak (1988) presented a method where individuals were divided into groups, and if the groups were significantly different then each one would have a separate matrix. On the other hand in Basak and Saaty (1993) the authors divided the individuals into a number of groups. Then the authors used the likelihood-ratio-test criterion for testing the hypothesis if the group judgments were homogeneous or significantly different. They also conducted a comparison based on 27 and 10 individuals divided into two groups. However, it was not specified how to divide the individuals into the specific groups. The geometric or arithmetic mean are mainly used to average the assessed weights or preferences from different decision makers (Lin and Lu 2012). Aczel and Saaty (1983) and Basak and Saaty (1993) showed for instance that the geometric mean is a good way to aggregate expert scores when equal importance is to be given to the experts in a group. However, Aczel and Saaty (1983) did not justify when to combine expert judgments. Lin and Lu (2012) stated that by using average-type manipulations the variations or dispersion among the experts is usually ignored, and that the manipulations are vulnerable to extreme values.

Therefore in this study, the scores from the experts were aggregated into three scoring sets, i.e., first quartile Q1, second quartile Q2, and third quartile Q3 instead of using the statistical methods mentioned above. By using the first quartile, the lowest 25 of the scores were split from the highest 75%, or in other words where 25% of the scores was less than the calculated Q1 value. Similarly, the second quartile was the median of scores, and third quartile marked the value where 75% of the data was less than the calculated Q3 value. By using these three methods of aggregating the expert scores it was possible to analyze the effect of how this type of aggregation (i.e., distortion) would affect the susceptibility, and if consistency could be achieved after aggregating the individual inconsistent judgments.

When aggregating the scores according to Q1 more influence was given to the experts that scored lower than the rest of experts. Similarly, when aggregating the scores according to Q3 more influence was given to the experts that scored higher than the rest. When using Q2, or the median to split scoring sets, of scores for each perspective it was possible to encompass the whole scoring range, i.e., not giving a priority to those experts that scored higher or lower than the others. However, aggregating the different expert scorings into one scoring set could weaken the overall scoring and cause inconsistency (*CR> 0.10*). In this study a *CR* over the recommended 0.10 was seen for the perspectives inundation Q2 (*CR* of 0.12), inundation Q3 (*CR* of 0.13), landslide Q3 (*CR* of 0.14), debris flow Q3 (*CR* of 0.20). This indicated that the scores calculated in Q1 were consistent for inundation whereas the two other aggregation methods were not, and thus Q1 would be the most appropriate scoring set used for the susceptibility assessments for any perspective. Q3 resulted in inconsistency for all perspectives, and thus should be considered the least appropriate aggregation set.

Lin and Lu (2012) stated that the measure of consistency in AHP is an important aspect but it is not the main goal of decision making. Consistency is however an important criterion for the acceptance of an expert’s judgments. Therefore, in order to achieve a consistency in the scoring (i.e., *CR* < 0.10) for the three perspectives in the future, the experts should be asked to participate in a workshop where they first screen for important factors and score them individually. This would also help those experts that might be unfamiliar with some specific factor, such as the TWI which was the case for two experts in this study. A second step in the workshop could be that the experts get to score the factors as a group for instance by using the Delphi method in order to discuss and agree on the most appropriate score. This would help in order to improve logical consistency (Banai 2006). For a susceptibility assessment it is desirable, but not always possible, to have several experts from different fields scoring the factors. For instance, in Lin and Lu (2012) 18 decision makers participated in their pairwise comparison evaluation, in Oswald Beiler and Treat (2015) 12 transportation practitioners participated in their survey, and in Sahin et al. (2013) 33 questionnaires were answered out of the 75 sent out. However, in Banai (2006) it was not stated how many participants were included in their study.

As mentioned previously, only eight experts replied in this study. This is a small cross-section of experts (8 out of 20, or 40% return rate) too few for the answers to be considered statistically significant. Although small cross-section it is not entirely different from other studies, see for instance Sahin et al. (2013) where their return rate was 44%. Experience from STA staff and from several road-climate related seminars also evidenced that the number of Swedish experts working with climate-change and natural hazards aspects on road planning is hitherto low (Kalantari and Folkeson 2013). Sahin et al. (2013) stated that the AHP is not a statistical method but a subjective method. Therefore, it is not necessary to involve a large sample, as a large sample might cause impracticality where the participants could have a tendency to provide arbitrary answers. This could in the end cause a very high degree of inconsistency. They also stated that a smaller sample size is adequate enough for implementing the AHP. The most important is whether or not the sample size reflects the opinions of all the stakeholders. The variation in the scores provided by the experts in this study indicated the difficulty of pair-wise comparison between the factors, and at the same time to fully understand the importance of the factors on the perspective itself.

#### WLC, equal weighting, and FIM

As mentioned previously, WLC is one of the most common decision rules. Its applicability for spatial suitability analysis is broad as it is easy implemented in GIS. Because of this it is likely to be chosen as the primary method when decision makers and planners are facing a decision problem. However, even if WLC is easy to implement the results can be uncertain (Malczewski 2000). This could be the case if analysts for instance ignore or are unaware of the assumptions taken and apply the WLC incorrectly. One of the most important aspects of WLC is the weighting process, as it is a central step in obtaining the decision maker’s preferences. It is also an especially common error when applying WLC to spatial decision problems (Malczewski 2000). However, in WLC a low score on one criterion, i.e., low weight, can be compensated or traded-off by a high score on an another criterion (Karlson et al. 2016). This could be considered a negative aspect of WLC if the criteria chosen should not be traded-off. Ideally, factors in additive models such as WLC or any other multi-criteria analysis should be independent of each other (Munda 2008). Therefore, WLC should also only be used to aggregate strongly independent criteria. Although, many of the factors used in this study can be considered to be interlinked in nature and would have an effect on each other, such as the factor soil thickness which has an influence on the land cover, or the land cover which influences the slope in such a way that the land cover stabilizes the slope and reduces erosion, it was mentioned in Karlson et al. (2016) that factors and criteria indicate different aspects of suitability or susceptibility; which might not necessarily correlate and result in double counting. Malczewski (2000) also stated that the requirement of decomposability and non-redundancy are very difficult to satisfy for spatial decision problems, as some pairs of attributes will be correlated. This redundancy could be avoided by using surrogate attributes which are to replace the overlapping pairs or subset of attributes. However, in this study, a third method i.e., *FIM* was used in order to account for the linkages, i.e., interdependence, between the factors.

By using the *Equal weighting* scheme for the susceptibility assessment all factors were given the same influence for the susceptibility to inundation, landslide and debris flow. The difference between the three perspectives could only be noticed through the factors internal classes, ranking and standardization (Table 2 and Table 3). The resulting susceptibility for inundation using the *Equal weighting* was higher than any other decision rule for the entire area and the three road objects. For the failure points it performed similarly to all other decision rules except for *AHP Q1* (Fig. 12b, Table 10). Downscaling into the four failure points largely affected the percentage of susceptibility using the *AHP Q1* weighting set. Using this weighting set would suggest that the four failure points would not be susceptible. However, these are the four points where road embankment failure actually did occur. Thus, using this aggregation set for decision support would be highly misleading.

Considering the difference between the highest susceptibility results from *AHP Q1, Q2,* and *Q3* and using the *Equal weighting* scheme (Table 10) it could be seen that the overall difference between using and not using expert judgment for weighting was small (average around 4% and maximum difference 10% for inundation).

Using *Equal weighting* could be considered as a neutral weighting scheme in many cases, where the actual influence of each criterion to the actual perspective is ignored. However, for this study it was used as a reference point (all criteria are treated the same) in order to be able to analyze the effect of both expert judgment, aggregation of expert judgments and the *FIM*. In order to account for the redundancy between the criteria used, the *FIM* method was employed. This method accounts for the influence between the criteria and give more weight to the more influential ones (Fig. 8 and Table 9). The less influence a criterion has the less weight and thus less contribution it has to the susceptibility assessment. In this study both slope and land cover was considered equally important, whereas distance to stream and distance to lake were considered the least important. Using *FIM* resulted in highest *Class 2* percentage of all decision rules for the entire area (Fig. 10) and generally close to the performances of the downscaled subsets of the study area using the *Equal weighting* scheme.

A method that could be used to account for interdependence between factors is for instance the analytical network process (ANP) (Saaty 1999). The main difference is that ANP uses a network instead of a hierarchy. This feature is in its advantage since many decision problems cannot be structured hierarchically due to the interdependence of higher-level elements in a hierarchy (Saaty 2008b). In ANP composite priority ratio scales are derived from individual ratio scales which represent relative measurements of the influence of elements that interact with respect to control criteria (Saaty 1999). Saaty (2008b) stated that the world is far more interdependent than we know how to deal with using our existing ways of thinking and acting, and that ANP is the logical way in how to deal with this dependency. In AHP the decision problem is represented by a network consisting of nodes. In this study FIM was used as it is a relatively simple version of a network process and fairly straight-forward. However, it needs control for circularity. In future method development, the importance of the factors as well as their interdependencies could be integrated in order to take both aspects into account simultaneously.

### Selection of Factors, Constraints, and Fuzzy Values

The selection of factors and constraints must be appropriate for the objective. Adding too many factors and constrains will ultimately result in a more complex system. A limit of 7 ± 2 criteria serves also a practical purpose as this criteria limit avoids confusion in paired comparisons when the criteria are considered simultaneously (Banai 2006). In addition, many SMCA studies of localization problems also use constraints to exclude areas from suitability considerations (e.g., Karlson et al. 2016). However, since the objective of this study was to assess the susceptibility for natural hazards, the use of constraints was not considered to be applicable. In this study the selection and the number of factors was based on previous studies and study area characteristics (See section 2.2), and where finding areas susceptible to inundation, landslide or debris flow was the objective. Although the three perspectives could have different factors among them, including factors concerning economy, ecology, and society, many of the geological factors have been found to be the same. Some of these factors are soil thickness, geology (which encompass soil type and rock outcrops), slope, distance to streams and lakes, and TWI. Some of these are also factors which should be tangible to experts and thus enable an understanding of the coupling between the factors and perspectives.

For instance, for landslide assessment the importance of the factor land cover lays within the environmental conditions for slope failure. The vegetation cover and the removal of the vegetation are factors which can contribute to instability by either governing the movement of a slope or dictate the conditions of the movement (Glade 1998; Feizizadeh and Blaschke 2013). Deforestation is also one of the main causes for the change in flood risk of an area (Kourgialas and Karatzas 2011). Kalantari et al. (2014b) found that flood probability was positively correlated with the percentage of grassland but negatively correlated with forest in similar Swedish settings to those considered in the current study. Therefore it could be expected that catchments with a large proportion of mature forest and tree-canopy interception decrease the probability of flooding of a road located downslope as well as the susceptibility to either landslide or debris flow. The geology or soil type can cause an area to become more susceptible to flood inundation (Saha et al. 2005). It also affects the speed of hydrological response in a catchment (Kalantari et al. 2014b).

For the landslide susceptibility assessment the geology (i.e., soil type) was also important to consider since it affects the characteristics of the landslide. Permeable soils such as gravel and coarse sand with higher infiltration capacity also reduce the probability of inundation. Soils with lower hydraulic conductivity such as silt and peat increase the probability of inundation. One of the important factors to be taken into consideration for inundation assessment is the storage capacity of the soils, which is dependent on soil thickness, porosity and initial soil moisture (Kutilek and Nielsen 1994). If the soil does not have a sufficient storage capacity due to shallowness and/or having high initial moisture content, most of the rainfall will contribute to runoff generation. The soil thickness and the infiltration of rainwater in slopes is an important aspect to consider when assessing the slope-stability, especially for high intensity rainfall events (Mukhlisin et al. 2008; Ho et al. 2012). Mukhlisin et al. (2008) concluded that shallower soils increased the discharge volume, reduced the peak pore water pressure and thus also reduced the probability of slope failure; whereas deeper soil thickness increased the weight of the solids, soil moisture content and pore water pressure which resulted in slope failure.

In this study a soil thickness model developed by SGU was used. This model is known to have uncertainties for sparsely populated areas which tend to have low data availability (Karlsson et al. 2014). Therefore the susceptibility assessment would be influenced by this criterion and its inherited uncertainties. Further the slope angle is an important factor both for flood and landslide assessments. Locations with equivalent slopes but larger upslope drainage areas have an increased risk for flooding during extreme conditions such as heavy rainfall events (Kalantari et al. 2014b). The slope angle also influences the water transfer rate between the various soil layers; a steeper slope reduces the risk for flooding. For land sliding the slope angle influences the susceptibility, where increased angles are correlated with the increased likelihood of slope failure (Dai and Lee 2002).

TWI was chosen as a factor in this susceptibility assessment because of its representation of the water accumulation in a catchment and thus the capability of any point in the area to develop saturated conditions. It also is important to consider during landslide assessments since soil saturation can trigger landslide events. Considering the straightforward equation used in GIS it can be noted that the TWI does not account for the soil type or land cover, both of which are affecting the water saturation in an area. This was accounted for when using *FIM*, however it was not accounted for directly in AHP but indirectly through the overlay procedure in GIS.

### Uncertainties in the Susceptibility Assessment and Use for Decision Support

The susceptibility assessment was subjected to various uncertainties due to the nature of the model itself. The assessment was based on more or less subjective, and partly inconsistent, expert judgments which increased the uncertainty. In addition, many assumptions had to be made, for example on the ranking of criteria classes, on the fuzzy membership functions and thresholds, as well as on the break values between low susceptibility and high susceptibility. All these assumptions would affect the overall susceptibility assessment.

High uncertainties also lie in the break values used for dividing the results into high and low susceptibility. More studies would be needed to develop guidelines as to what should be considered high or low susceptibility, which would be very useful for road planning. In this study the differences between the data values were used for data clustering through natural breaks. Any change in the way break values are selected for dividing the results into classes would affect the percentage of *Class 1* and *Class 2* susceptibility. In reality nature is of course very complex, and areas assessed as highly susceptible in an assessment might never experience a failure whereas areas classed as low susceptible might do. Therefore it is important to recognize the uncertainty in the resulting susceptibility assessment, especially if it will be used for decision support.

In its current form, the SMCA approach for susceptibility assessment can be seen as a spatial regional or landscape scale guide considering the criteria selected for the analysis. Even though the cross-section of experts in this study was small, the study demonstrates that expert opinion can vary significantly which underlines the need for a systematic methodology to integrate across various opinions and fields of knowledge. Still, susceptibility assessment through SMCA is a useful method for decision support in early stages of road planning, even though it needs further development concerning e.g., the decision rules and the criteria. A natural hazard susceptibility SMCA could be used to indicate areas where more investigations need to be undertaken from inundation, landslide or debris flow points of view. It could also be used to identify areas thought to have higher susceptibility along existing roads and if needed where mitigation measures could be target after in-situ investigations.

## Conclusion

The susceptibility assessments through SMCA and different decision rules showed that inundation was the perspective that resulted in the highest percentage of susceptibility. By aggregating the individual expert judgments it was possible to reach a consistency in the aggregated scoring sets despite the fact that many individual sets were inconsistent from the beginning. Consistency could be reached for all perspectives when the scores were aggregated so that the weights for susceptibility would be influenced by the lower range of expert scores. Using expert judgment for scoring through the Saaty scale of importance and *AHP* showed to perform almost the same as *Equal weighting*. By accounting for the influence between the criteria *FIM* was used, and the susceptibility using this method resulted in the highest susceptibility for all three perspectives. This method could be considered to over-dimension the susceptibility compared to the other methods. The susceptibility using aggregated score AHP on the other hand resulted in lower susceptibility compared to the other decision rules. When downscaling was undertaken into the four failure points that experienced road embankment failures, the percentage of susceptibility using *AHP* and expert scores in the lower range for deriving the weighting set was largely affected in a positive manner. Therefore using this weighting set would suggest that the four failure points would not be susceptible, so if this aggregation set was used for decision support it would be highly misleading.

The susceptibility SMCA needs further development concerning e.g., decision rules and criteria. However, despite the current limitations of SMCA it is still a useful method for spatially identifying potentially natural hazard susceptible areas considering the used criteria, but it should be followed up with in-situ investigations and more detailed modeling for use as decision support for early road planning. The natural hazard susceptibility SMCA could be used to indicate areas where more investigations need to be undertaken from inundation, landslide or debris flow points of view. It could also be used to identify areas thought to have higher susceptibility along existing roads and if needed where mitigation measures could be target after in-situ investigations. In sum, susceptibility assessment SMCA is a useful method for decision support in early stages of road planning.

## Appendix A

Table 11

**Table 11:** Natural break values for division into susceptibility classes

Inundation		
AHP individual experts	Class 1	Class 2
Expert 1	186	252
Expert 2	171	250
Expert 3	201	255
Expert 4	168	250
Expert 5	176	253
Expert 6	175	243
Expert 7	175	243
Expert 8	179	246

AHP aggregated	Class 1	Class 2
AHP Q1	182	246
AHP Q2	162	248
AHP Q3	164	250

WLC	Class 1	Class 2
Equal weighting	151	244

WLC	Class 1	Class 2
FIM	138	245

Landslide		
AHP individual experts	Class 1	Class 2
Expert 1	203	255
Expert 2	193	255
Expert 3	202	255
Expert 4	206	255
Expert 5	195	255
Expert 6	207	255
Expert 7	180	255
Expert 8	186	255

AHP aggregated	Class 1	Class 2
AHP Q1	199	255
AHP Q2	197	255
AHP Q3	196	255

WLC	Class 1	Class 2
Equal weighting	201	255

WLC	Class 1	Class 2
FIM	202	255

Debris flow		
AHP individual experts	Class 1	Class 2
Expert 1	198	255
Expert 2	198	255
Expert 3	202	255
Expert 4	195	255
Expert 5	202	255
Expert 6	204	255
Expert 7	178	255
Expert 8	184	255

AHP aggregated	Class 1	Class 2
AHP Q1	205	255
AHP Q2	201	255
AHP Q3	198	255

WLC	Class 1	Class 2
Equal weighting	201	255

WLC	Class 1	Class 2
FIM	196	255

## Appendix B

Table 12

**Table 12:** Individual expert scoring according to the Saaty scale

Expert 1							
Inundation (CR: 0.11)	Slope	Geology	Land cover	Soil thickness	Distance to streams	Distance to lakes	TWI
Slope							
Geology	1/3						
Land cover	1/3	1					
Soil thickness	1/7	1	1				
Distance to streams	9	9	9	9			
Distance to lakes	7	7	7	7	1/5		
TWI	3	3	3	3	1/7	1/7	

Landslide (CR: 0.10)	Slope	Geology	Land cover	Soil thickness	Distance to streams	Distance to lakes	TWI
Slope							
Geology	3						
Land cover	1/3	1/7					
Soil thickness	1/3	1/7	1/3				
Distance to streams	1/7	1/7	1/3	3			
Distance to lakes	1/7	1/9	1/3	3	1		
TWI	1/3	1/5	1	1	1	5	

Debris flow (CR: 0.03)	Slope	Geology	Land cover	Soil thickness	Distance to streams	Distance to lakes	TWI
Slope							
Geology	1						
Land cover	1	1					
Soil thickness	1/5	1/5	1/5				
Distance to streams	1/7	1/7	1/7	1/3			
Distance to lakes	1/7	1/7	1/7	1/3	1		
TWI	1	1	3	7	7	7	

Expert 2							
Inundation (CR: 0.15)	Slope	Geology	Land cover	Soil thickness	Distance to streams	Distance to lakes	TWI
Slope							
Geology	1/3						
Land cover	1/3	3					
Soil thickness	1/7	1/3	1/7				
Distance to streams	3	1	1	5			
Distance to lakes	3	1	1	5	1		
TWI	3	1	1	7	1/3	1/3	

Landslide (CR: 0.13)	Slope	Geology	Land cover	Soil thickness	Distance to streams	Distance to lakes	TWI
Slope							
Geology	1						
Land cover	1/7	1/5					
Soil thickness	1/3	1/3	1				
Distance to streams	1	1	3	1			
Distance to lakes	1	1	3	1	1		
TWI	3	1/3	1	1	1/3	1/3	

Debris flow (CR: 0.13)	Slope	Geology	Land cover	Soil thickness	Distance to streams	Distance to lakes	TWI
Slope							
Geology	1/3						
Land cover	1/3	1					
Soil thickness	1/5	1/3	1/7				
Distance to streams	1/3	1	1/5	1			
Distance to lakes	1/9	1/9	1/9	1/5	1/9		
TWI	1	1	1	1	1/3	3	

Expert 3							
Inundation (CR: 0.29)	Slope	Geology	Land cover	Soil thickness	Distance to streams	Distance to lakes	TWI
Slope							
Geology	1						
Land cover	3	1/5					
Soil thickness	1/3	1	3				
Distance to streams	1	1/5	3	1			
Distance to lakes	1/3	1/5	3	1	1/3		
TWI	1/3	3	3	1	3	1	

Landslide (CR: 0.29)	Slope	Geology	Land cover	Soil thickness	Distance to streams	Distance to lakes	TWI
Slope							
Geology	1						
Land cover	3	1/5					
Soil thickness	1/3	1	3				
Distance to streams	1	1/5	3	1			
Distance to lakes	1/3	1/5	3	1	1/3		
TWI	1/3	3	3	1	3	1	

Debris flow (CR: 0.14)	Slope	Geology	Land cover	Soil thickness	Distance to streams	Distance to lakes	TWI
Slope							
Geology	1/3						
Land cover	1/3	1/3					
Soil thickness	1/5	1	3				
Distance to streams	1/3	1/5	3	1			
Distance to lakes	1/5	1/5	3	1	1/3		
TWI	1/3	3	3	1	3	1	

Expert 4							
Inundation (CR: 0.98)	Slope	Geology	Land cover	Soil thickness	Distance to streams	Distance to lakes	TWI
Slope							
Geology	9						
Land cover	1/9	1/9					
Soil thickness	1/5	1/7	1/7				
Distance to streams	3	1/9	1/9	1/5			
Distance to lakes	3	1/9	1/9	1/9	1		
TWI	n/a	n/a	n/a	n/a	n/a	n/a	

Landslide (CR: 0.91)	Slope	Geology	Land cover	Soil thickness	Distance to streams	Distance to lakes	TWI
Slope							
Geology	3						
Land cover	1/9	1/9					
Soil thickness	1/5	1/7	1/7				
Distance to streams	3	1/9	1/9	1/5			
Distance to lakes	3	1/9	1/9	1/5	1		
TWI	n/a	n/a	n/a	n/a	n/a	n/a	

Debris flow (CR: 1.57)	Slope	Geology	Land cover	Soil thickness	Distance to streams	Distance to lakes	TWI
Slope							
Geology	1/5						
Land cover	1/3	1/9					
Soil thickness	3	1/7	1/7				
Distance to streams	9	1/9	1/9	1/5			
Distance to lakes	9	1/9	1/9	1/9	1		
TWI	n/a	n/a	n/a	n/a	n/a	n/a	

Expert 5							
Inundation (CR: 0.03)	Slope	Geology	Land cover	Soil thickness	Distance to streams	Distance to lakes	TWI
Slope							
Geology	1/3						
Land cover	1/5	1/3					
Soil thickness	1/7	1/3	1				
Distance to streams	1	3	5	7			
Distance to lakes	1	3	5	7	1/3		
TWI	n/a	n/a	n/a	n/a	n/a	n/a	

Landslide (CR: 0.05)	Slope	Geology	Land cover	Soil thickness	Distance to streams	Distance to lakes	TWI
Slope							
Geology	1/2						
Land cover	1/7	1/7					
Soil thickness	1	1/3	5				
Distance to streams	1/9	1/5	3	1/3			
Distance to lakes	1/9	1/7	1	1/5	1/2		
TWI	n/a	n/a	n/a	n/a	n/a	n/a	

Debris flow (CR: 0.04)	Slope	Geology	Land cover	Soil thickness	Distance to streams	Distance to lakes	TWI
Slope							
Geology	1						
Land cover	1/5	1/5					
Soil thickness	1/5	1/3	3				
Distance to streams	1/3	1/5	2	1/3			
Distance to lakes	1/3	1/5	2	1/2	1		
TWI	n/a	n/a	n/a	n/a	n/a	n/a	

Expert 6							
Inundation (CR: 0.19)	Slope	Geology	Land cover	Soil thickness	Distance to streams	Distance to lakes	TWI
Slope							
Geology	1						
Land cover	1	1					
Soil thickness	1	1	1/9				
Distance to streams	1/9	1	1	1			
Distance to lakes	1/9	1	1	1	1		
TWI	9	9	9	9	9	9	

Landslide (CR: 0.19)	Slope	Geology	Land cover	Soil thickness	Distance to streams	Distance to lakes	TWI
Slope							
Geology	1						
Land cover	1	1					
Soil thickness	1	1	1/9				
Distance to streams	1/9	1	1	1			
Distance to lakes	1/9	1	1	1	1		
TWI	9	9	9	9	9	9	

Debris flow (CR: 0.08)	Slope	Geology	Land cover	Soil thickness	Distance to streams	Distance to lakes	TWI
Slope							
Geology	1						
Land cover	1	1					
Soil thickness	1	1	1/9				
Distance to streams	1	1	1	1			
Distance to lakes	1	1	1	1	1		
TWI	9	9	9	9	9	9	

Expert 7							
Inundation (CR: 0.08)	Slope	Geology	Land cover	Soil thickness	Distance to streams	Distance to lakes	TWI
Slope							
Geology	1/7						
Land cover	1/5	1/3					
Soil thickness	1/7	1	1/3				
Distance to streams	1/5	3	1	1			
Distance to lakes	1/5	3	1	1	1		
TWI	1/5	1	1	1	1	1	

Landslide (CR: 0.13)	Slope	Geology	Land cover	Soil thickness	Distance to streams	Distance to lakes	TWI
Slope							
Geology	1/9						
Land cover	1/5	1/3					
Soil thickness	1/3	1/3	1/5				
Distance to streams	1/7	1/5	1/5	1/3			
Distance to lakes	1/7	1/5	1/5	1	1		
TWI	1/3	1/3	1/5	1	1	1	

Debris flow (CR: 0.12)	Slope	Geology	Land cover	Soil thickness	Distance to streams	Distance to lakes	TWI
Slope							
Geology	1/7						
Land cover	1/7	1/3					
Soil thickness	1/3	1/5	1/3				
Distance to streams	1/5	1/5	1	1			
Distance to lakes	1/5	1/5	1	1	1		
TWI	1/3	1	1	7	7	7	

Expert 8							
Inundation (CR: 0.21)	Slope	Geology	Land cover	Soil thickness	Distance to streams	Distance to lakes	TWI
Slope							
Geology	1/3						
Land cover	1/5	1/3					
Soil thickness	1/5	3	3				
Distance to streams	7	7	7	7			
Distance to lakes	5	5	5	5	3		
TWI	1	3	3	3	3	3	

Landslide (CR: 0.10)	Slope	Geology	Land cover	Soil thickness	Distance to streams	Distance to lakes	TWI
Slope							
Geology	1/5						
Land cover	1/5	1/3					
Soil thickness	1/3	1/3	3				
Distance to streams	1/7	1/3	1/3	1/5			
Distance to lakes	1/7	1/5	1/3	1/5	1		
TWI	1/3	1/3	3	1/5	3	5	

Debris flow (CR: 0.12)	Slope	Geology	Land cover	Soil thickness	Distance to streams	Distance to lakes	TWI
Slope							
Geology	1/5						
Land cover	1/7	1/5					
Soil thickness	1/3	1	3				
Distance to streams	1/5	1/5	1/5	1/5			
Distance to lakes	1/7	1/7	1/7	1/7	1/3		
TWI	1/5	1/3	1/5	1/5	1	1	
